# Post-COVID-19 WHO Reform: Ethical Considerations

**DOI:** 10.1093/phe/phab011

**Published:** 2021-05-19

**Authors:** Thana C de Campos-Rudinsky

**Affiliations:** School of Government, Pontificia Universidad Catolica de Chile, Von Hügel Institute, University of Cambridge, and UNESCO Chair in Bioethics and Human Rights

## Abstract

This study argues against the expansive approach to the WHO reform, according to which to be a better global health leader, WHO should do more, be given more power and financial resources, have more operational capacities, and have more teeth by introducing more coercive monitoring and compliance mechanisms to its IHR. The expansive approach is a political problem, whose root cause lies in ethics: WHO’s political overambition is grounded on WHO’s lack of conceptual clarity on what good leadership means and what health (as a human right) means. This study presents this ethical analysis by putting forth an alternative: the humble approach to the WHO reform. It argues that to be a better leader, WHO should do much less and have a much narrower mandate. More specifically, WHO should focus exclusively on coordination efforts, by ensuring truthful, evidence-based, consistent, and timely shared communications regarding PHEIC among WHO member-states and other global health stakeholders, if the organization desires to be a real global health leader whose authority the international community respects and whose guidance people trust.

## Introduction

The COVID-19 pandemic has revived the protracted political debates on the need to reform the World Health Organization (WHO).^1^ These debates tend to focus on the political implications of said reform, arguably because of their more assessable impacts. The ethical considerations of how the WHO should be reformed to fulfill its purpose well are often left aside, as global health experts tend to perceive theoretical clarifications as less immediately relevant. This study provides a moral critique of the main proposals for WHO reform, currently under debate in light of the COVID-19 pandemic. In this way, this study fills a knowledge gap in the WHO reform literature, by introducing the perspective of ethics to critically examine the WHO mandate. Global health scholars conventionally claim that the WHO’s mandate should be reformed by way of expansion. In other words, they argue that, in order to be a better global health leader, WHO should do more, be given more power and financial resources, have more presence on the ground (where outbreaks actually happen), and have more teeth by introducing more coercive monitoring and compliance mechanisms to its International Health Regulations (IHR)^2^—the binding legal instrument that regulates and coordinates the actions of WHO member-states in the event of public health emergencies of international concern (PHEIC) (Gostin, 2014, 2020; Gostin and Friedman, 2014; Gostin *et al.*, 2015; Kickbusch and Reddy, 2015; Mackey, 2016; Negri, 2018; Burci, 2020; Gostin and Wetter, 2020). I call this the expansive approach to WHO reform. This study evaluates this conventional approach in order to reveal the misunderstood correlation between being a good leader and having an ambitious mandate.

I argue that to be a better leader, WHO should do much less and have a much narrower mandate. More specifically, WHO should delegate more functions and tasks to other global health stakeholders (especially local actors), which are better situated to perform these functions and tasks in more effective and efficient ways. In delegating more functions and tasks to other global health stakeholders, WHO could free itself to focus the use of its scarce resources on coordination efforts, by ensuring, more specifically, that communications regarding PHEIC among all of these stakeholders are truthful, evidence-based, consistent, and timely shared. Gathering scientific evidence on how to control PHEIC and communicating it in a truthful and timely manner should be, I contend, WHO’s sole priority mission resulting from the reform process to follow COVID-19. There are two main reasons for this narrow mandate. First, by focusing exclusively on ensuring truthful and timely communications among stakeholders, WHO could be able to concentrate its finite capacities in performing well its key function as a coordinating body for global health threats like pandemics. Second, in performing this core purpose well, WHO could potentially regain public trust as a legitimate coordinating authority for global health security matters. Perhaps once trustworthiness is recovered, WHO could then try to perform additional tasks and functions—if the international community judges this expansion suitable. However, the priority for the moment is, I suggest in this study, to tame WHO’s ambitious mandate with prudence and hindsight.

Although WHO’s overambition is a political problem, its root cause lies in ethics: WHO’s political overambition is grounded on WHO’s lack of conceptual clarity on what good leadership means and what health (as a human right) means. This study presents this ethical analysis in the following way: first it will discuss the expansive approach to the WHO reform and how it conflates good leadership in global health governance with the ambition (perhaps desirable yet not politically feasible or morally justifiable) to attain for all peoples the (impossible) standard of complete health, as defined in the WHO constitution and United Nations documents establishing the human right to health. Then, the study goes on to justify why WHO should be less ambitious, do less, and have a much narrower mandate (i.e., focused on good coordination by ensuring truthful, evidence-based, consistent, and timely shared communications regarding PHEIC among WHO member-states and other global health stakeholders) if the organization desires to be a real global health leader whose authority the international community respects and whose guidance people trust.

## The Expansive Approach to WHO Reform

The WHO was founded in the aftermath of the Second World War, in 1948, as an international organizational and specialized agency of the United Nations, for the “the purpose of co-operation to promote and protect the health of all peoples”.^3^ Article 1 of the WHO constitution specifies the objective of the WHO as the coordinating authority for global health in this way: “the objective of the WHO shall be the attainment by all peoples of the highest possible levels of health,” where health is, “a state of complete physical, mental and social well-being and not merely the absence of disease or infirmity”.^4^ In order to attain this ambitious objective, the WHO constitution then lists, in its article 2, a lengthy list of 22 functions that the WHO ought to perform as the coordinating authority for global health. These include, for example, certain functions that are more directly relevant in the context of pandemics, such as the function (g) to stimulate and advance work to eradicate epidemic, endemic, and other diseases; (k) to propose conventions, agreements and regulations, and make recommendations with respect to international health matters; and (s) to establish and revise as necessary international nomenclatures of diseases, of causes of death and public health practices. However, article 2 of the WHO constitution also includes a number of more general functions that the organization is equally expected to perform to contribute to well-being broadly defined. For example, WHO has also (i) to promote, in cooperation with other specialized agencies where necessary, the improvement of nutrition, housing, sanitation, recreation, economic or working conditions and other aspects of environmental hygiene. The WHO today call these the “Social Determinants of Health”—SDH:

the conditions in which people are born, grow, work, live, and age, and the wider set of forces and systems shaping the conditions of daily life. These forces and systems include economic policies and systems, development agendas, social norms, social policies and political systems.^5^

In providing “a comprehensive blueprint for human development,” SDH includes both conditions that are directly connected to health and conditions that are more tangentially connected to health. Either direct or tangential, these are all surely important.^6^ Some of them are considered so important that they ground separate rights (e.g., right to housing, right to work, and environmental rights), which presumably would equally be part of WHO’s mandate by way of conceptually falling under the SDH’s comprehensive category. What these general functions of the WHO and the SDH evince is the all-inclusiveness of WHO’s functions. To be clear, WHO’s functions are not restricted to controlling communicable disease outbreaks but go well beyond it in order to respond to WHO’s ambitious mandate of attaining the highest levels of complete physical, mental and social well-being for all people.

### The Politics Behind WHO’s Ambition

One reason for WHO’s ambition is political. WHO’s mandate and functions, as defined in WHO’s constitution, have continually stretched over the past decades. With the acceleration of globalization in the 1990s, there has been a proliferation of global health initiatives (e.g., the United Nations Joint Programme on HIV/AIDS, Global Alliance on Vaccines and Immunization, the Global Fund to Fight HIV/AIDS, Tuberculosis and Malaria, the International AIDS Vaccine Initiative, the Medicines for Malaria Venture, the US President’s Emergency Plan for AIDS Relief, the UK Department for International Development, the Bill and Melinda Gates Foundation, to name just a few). What this meant is that, by the year 2000, WHO was competing with all of these new global health institutions for funding (Lee and Pang, 2014: 120). The upshot of this highly competitive scenario was that WHO lost the autonomy to define its own institutional priorities: donors now stipulate how the capital they invest in the WHO should be spent (Liden, 2014: 142). In other words, donors’ multiple interests, instead of global health needs, now determine WHO’s priorities. Donors’ interests are numerous, ranging from pandemic preparedness and response to noncommunicable diseases, such as obesity and mental health, to reproductive rights, to regulation of tobacco use, to poverty-related illnesses and social justice, to traffic accidents, to air pollution and environmental issues, and so on. No one would question the importance of these matters: these are the vast majority of the global burden of disease. It would seem therefore fitting that they should all fall under the WHO’s remit. However, this plethora of interests that now defines WHO’s priorities are causing confusion when it comes to choosing between priorities, to such an extent that WHO itself has recognized that “it has too many priorities, everything being a priority” (WHO, 2013a: 16).

Although WHO has recognized its limitations by self-proclaiming itself “overcommitted, overextended and in need of reform” (WHO, 2013b: para 50), the most recent WHO reforms have ironically been geared toward further expanding WHO’s commitments. For example, in the aftermath of the Ebola outbreak of 2014, the WHO implemented a number of reforms, including programmatic as well as institutional changes (Moon *et al.*, 2017). Global health experts argue that programmatic reforms have strengthened WHO’s operational capacity—meaning WHO’s ability to actually put “boots on the ground” so to speak, by creating a health emergencies program (the Global Outbreak and Response Network), as well as a contingency fund to provide expedited capital in emergencies, and a global health work force (Moon *et al.*, 2017). Global health scholars also continue to advocate for WHO’s institutional reforms, in particular those that will further strengthen WHO’s monitoring and compliance mechanisms (Moon *et al.*, 2017). These institutional reforms would necessitate, of course, an increase in resources: without additional funding there cannot be a proper implementation of such monitoring and compliance mechanisms (Moon *et al.*, 2017). For this reason, the US and Brazil’s recent announcements of their desire to withdraw from the WHO in the middle of the COVID-19 pandemic were met with intense criticism. (Burci, 2020; Gostin, 2020; Gostin and Wetter, 2020).

For the post-COVID-19 reforms, global health experts, by and large, continue to strongly push for the expansive approach to WHO reform. As supporters of the WHO, they acknowledge the limitations of WHO’s capacity in light of the currently available funding, and push for more funding for the organization. They claim, accordingly, that WHO should grow in capital, so that the organization can do more in terms of both operational capacity as well as monitoring and compliance (Burci, 2020; Gostin, 2020; Gostin and Wetter, 2020). For them, only if WHO is given more power and resources, can it become an effective global health leader (Gostin, 2020; Gostin and Wetter, 2020). Enhanced effectiveness in global health leadership would be the result of both more “boots on the ground” and more “teeth” through coercive monitoring and compliance mechanisms to be introduced to the IHR (Burci, 2020). A leadership based on both boots and teeth is, they argue, the most effective way of fulfilling WHO’s “purpose of co-operation to promote and protect the health of all peoples” ^7^. Without power and resources, operational practices, and a collective system to monitor compliance, the argument goes, WHO would never have the necessary means to implement even WHO’s most basic functions to control communicable disease outbreaks—let alone the overall mission of the organization, namely “the attainment by all peoples of the highest possible levels of health”.^8^ Implicit here is the idea that a good leader in global health governance should strive to do all it takes, in using boots and teeth, to fully realize the ambitious objective of the highest standard of complete health (i.e., “a state of complete physical, mental and social well-being and not merely the absence of disease or infirmity” ^9^) for all. Alternatively put, a good global health leader should be ambitious enough to fulfill the vast mandate entrusted to them and strive to realize all aspects of the comprehensive definition of the heath for all people under their leadership. This ambitious aspiration and attitude would be, according to most global health experts, not only politically justifiable but also the ethical thing to do: it would be morally wrong not to do enough.

### The Ethics Behind WHO’s Ambition

As the global health steward, it would be by and large blameworthy for WHO not to fulfill the mandate entrusted to them. However, it would also be ethically flawed to defend WHO’s ambitious mandate (and the expansive approach to WHO reform) without acknowledging the conceptual problems inherent to it as well as its practical consequences. Basically, the ethical error behind the WHO’s mandate and the expansive approach is moral conflation, leading to confusion in decision-making.

I have explained elsewhere the problem of moral conflation related to the well-being conception of the human right to health and how it leads to confusion in practical deliberations, in the following way. I presented and compared two scenarios. In the first, a patient who had been infected with the Ebola virus in their last trip to West Africa. Although the patient has a chance of survival, they are in urgent need of ZMapp, an experimental biopharmaceutical drug under development for Ebola virus disease. The second scenario brings a 16-year-old patient, suffering from clinical depression, who wishes for a rhinoplasty, mainly for cosmetic purposes, but also hoping to improve some mild respiratory problems. The teenager claims that their appearance has been undermining their confidence to the point of ruining their quality of life and well-being. While treatment with ZMapp in the first scenario will enhance the patient’s chances of survival, rhinoplasty in the second scenario may enhance the patient’s aesthetic sentiment of appreciation and perhaps their overall subjective emotional evaluation of their self, and therefore their quality of life and overall well-being.

The WHO’s duty to provide and the patient’s right to receive cosmetic surgery is *prima facie* and *ceteris paribus* less stringent than the WHO’s duty to provide and the patient’s right to receive medical treatment for the highly contagious and deadly Ebola disease. The latter is an obvious example of a *basic* health need. The former is not a *basic* health need; it is a nonbasic health need that nevertheless deserves government’s and society’s consideration since it may truly affect the patient’s mental health, quality of life, and overall well-being. Typically, basic health needs primarily involve matters of survival, which are *prima facie* and *ceteris paribus* far more morally stringent and urgent, in a scenario of allocation of scarce global healthcare resources, than minor risks to health, or in this case, aesthetic appreciation. However, when these two different scenarios are put together under the same label of the well-being conception of right to health, without an explicit distinction regarding their different priorities in a situation of scarce resources, the morally relevant distinction between basic and nonbasic health needs is obscured: the aspects of health that are basic for people’s survival, and all the remaining aspects of health that might affect the patient’s overall well-being are conflated.

The well-being conception of the human right to health does not clearly differentiate these different realities of health because both would be justified in attaining the general well-being of patients. As an alternative, I have proposed the basic health needs conception of the human right to health, which focuses on the central case of what constitutes the right to health (de Campos, 2017: ch 1). Basic health needs would include, for example, the provision of essential medication for the sick; the provision of basic healthcare infrastructure (including maternal, newborn, and child health care); sanitation (which is essential for preventing emerging pathogens, for containing outbreaks, and for responding to them when they start spreading); the provision of palliative care for the dying and those facing serious illness (de Campos, 2016: 75). This is, obviously, not meant to be a set list of basic health needs applicable to all populations equally: while, on the one hand, the basic health needs conception of the human right to health includes prophylactic treatments and therapeutic care that any human person objectively speaking need *prima facie* to survive, avoid a premature death, and allow for a well-managed death; on the other hand, the basic health needs conception of the human right to health also accounts for the fact that each person will in the reality of their unique circumstances necessitate these basic health needs in varying degrees and forms, depending on their context (de Campos, 2016: 75).

Notwithstanding, the reason why I mention here the well-being conception in contrast to the basic health needs conception of the human right to health is this: there is a need (not only in theory, for the sake of the moral precision in language, but also in practice, for the sake of confusion avoidance in decision making) to differentiate these two very distinct realities of health. And this is because different rights and duties bear on these two different aspects of global health justice, namely the basic and nonbasic needs of health, when one is dealing with priority-settings within a context of global scarcity (de Campos, 2016: 74).^10^ The stark contrast between the ZMapp and the rhinoplasty examples help illustrate these significant moral differences.

One may object here and claim that the well-being conception of the right to health is not necessarily inimical to priority setting. In this sense, my objector here would tell me that the conflation that I am worried about may not necessarily materialize, since the well-being conception of the right to health could make space for priority setting to be defined clearly. There is nothing in the well-being conception—my opponent would then conclude—that prevents priority setting.

It is perhaps true that the well-being conception of health would not make priority setting decisions utterly impossible. However, I would insist that the distinctions between basic and nonbasic should be made explicit, if confusion is to be avoided. Without clear distinctions between what is a basic priority and what is not, decisions on how the WHO should allocate scarce resources are made more difficult and more confusing than they should be—especially under very pressing scenarios, where WHO needs to make complex choices quickly, while facing pressure from different donors, pushing for different priorities for the organizations. To be more specific, these distinctions should be made explicit, if confusion is to be avoided, because the different rights and duties that bear on these two different aspects of global health justice, namely the basic and nonbasic needs of health, are not so straightforwardly discerned in practice—particularly within a very pressing context of global scarcity, coupled with WHO’s overcommitments and overextensions, where WHO is pulled in different directions. All of these make the distinction between priority and nonpriority a difficult and confusing enterprise for the WHO (WHO, 2013a: 16; WHO, 2013b: para 50).

The theoretical distinction between basic and nonbasic health needs is relevant not only for the theoretical purposes of philosophical contemplation but also for practical purposes of decision-making. Take, for example, the context of a catastrophic pandemic like COVID-19, where there has been much confusion about the priority allocation of scarce resources. Here conceptual clarity on the distinction between basic and nonbasic health needs would have been helpful in providing clear ethical justification and practical guidance for priority settings. The principles and reasons guiding the definitions of priorities should, however, be clearly stated before pressing decision-making occurs. Otherwise, confusion inevitable happens in the reality of complex real-world problems.

Now, even if one is convinced of the theoretical and practical necessities of distinguishing between basic and nonbasic health needs, one could still claim that perhaps the most complicated priority setting decisions are not so much between basic and nonbasic health needs, where the differences between priorities and nonpriorities are presumably clearer. Instead, the most complex decisions for the overcommitted and overextended WHO are actually between two basic health needs.

So let us compare these two scenarios. In the first, that same Ebola patient, who has a chance of survival, and is in urgent need of ZMapp. In the second scenario, a 33-year-old patient, suffering from colon cancer, who has a chance of survival, and is in urgent need of cancer treatment. Few would disagree that both ZMapp and cancer treatment are basic health needs, which will enhance the chances of survival of these two patients. But are WHO’s duty and capacity to provide ZMapp equal to WHO’s duty and capacity to provide cancer treatments? *Prima facie* and *ceteris paribus*, yes. But the problem is that things are not equal when it comes to WHO’s power and resources to provide both Ebola treatment and cancer treatment.

While both treatments are basic health needs, Ebola is considered an infectious disease that gained the status of PHEIC, and cancer is noncommunicable disease. PHEIC is therefore a sub-set of basic health needs that are serious and can quickly spread across political and geographical borders, to a point of possibly ensuing in a pandemic (de Campos, 2020). Undoubtedly, both Ebola and colon cancer are very serious illnesses that can cause excruciating suffering in patients. Ethically, both diseases therefore justify the need to be taken very seriously and to be addressed as urgently as possible by those with the capacity to address them most quickly and effectively. Now, while the WHO, as the chief coordinating body for global health threats, is in a unique position to address PHEIC quick and effectively, the organization has not been in a comparably advantageous position to address cancers of different sorts as quickly and effectively as other stakeholders (such as local or other global health actors who are better positioned or equipped to meet the basic health needs of local populations in need of cancer treatments of different sorts).

Arguably, the most commendable work that the WHO has done since its inception is on infectious diseases (Jha, 2017). This is because the WHO is uniquely placed to facilitate international coordination among different stakeholders, which is fundamental to contain the spread of epidemics worldwide. Focusing on PHEICs (which are, as I am defining here, a sub-set of basic health needs), while delegating the many other serious basic-health needs that arise outside of PHEIC (e.g., cancers) to other local and global health actors in a better position to address them efficiently and effectively with the inclusion of local communities is, I argue, justifiable for the purpose of WHO’s priority setting. To focus exclusively (at least for now) on the sub-set of basic health needs that qualify as PHEIC and to delegate the other basic health needs that are not PHEIC is practically and ethically reasonable. By differentiating these two types of basic health needs (i.e., those that are PHEIC and those that are serious noncommunicable diseases) and by arguing that only PHEIC should fall under WHO’s remit, I am not saying that PHEIC is more morally relevant than serious noncommunicable diseases. Nor am I saying that noncommunicable like cancers should not be a priority at all. Serious noncommunicable diseases are and should be a priority of local and global health stakeholders better equipped than the WHO to address these severely debilitating illnesses in an efficient and effective manner. The WHO, however, has never been in a position to do so well.

But my objector may want to contend here that it is not easy for WHO (or anyone) to choose between (i) treating the Ebola patient with some survival chances, or (ii) providing the cancer treatment to the young adult with equal or even higher chances of survival, or (iii) providing the rhinoplasty to the teenage patient who has an extremely debilitating appearance coupled with serious mental illness. My opponent here would argue that WHO should strive to provide *all of the three*, whenever possible. But I would here ask my opponent: is it possible? Has it ever been possible? Will it ever be possible? Global healthcare resources have always been scarce, so the ideal solution of providing Ebola treatment, and cancer treatment, and rhinoplasty is unworkable for the WHO. In fact, if Ebola ever becomes a pandemic, it is not even certain, based on our lived experience of COVID-19, that WHO would be able to ensure that all populations in all Nations could safely receive adequate Ebola treatment to contain the pandemic.

Now, I would also have to clarify here in response to my opponent that an unworkable situation does not automatically take away the ethical responsibilities of those involved. In fact, most ethical requirements are difficult to be put into practice and need consistent intention, good will, careful planning, and hard work. So, the fact that providing Ebola treatment, cancer treatment, and rhinoplasty is unfeasible does not automatically mean that providing all of them is not ethically required. So why then do I defend that providing all three is not ethically justifiable? Because in a context of scarce global healthcare resources, WHO has the ethical responsibility to make difficult choices and prioritize certain aspects of its mandate (i.e., PHEIC), while delegating some other aspects of its mandates to other global health stakeholders, namely governments, local communities, and other global health actors, which are better situated to address them. But this prudent delegation can only be fully ethically justified if the problem of moral conflation is fully appreciated.

To further explain the problem of moral conflation and examine how it leads to practical confusion when it comes to the specific theme of post-COVID-19 reforms of the WHO, I will return to the discussion of WHO’s 22 functions, listed in article 2 of the WHO constitution. Article 2 gives a good illustration of the problem of moral conflation when it includes in the same list very different aspects of health and well-being that will have to be considered within the reality of WHO’s scarce resources and limited capacities to fulfill them all. Take, for example, function (*g*) to stimulate and advance work to eradicate epidemic, endemic, and other diseases. Under function (g), one could think, more specifically, of ways in which WHO could, in coordination with other stakeholders, foster the dissemination of scientific data on the research and development of COVID-19 vaccines. A potential example here could include initiatives inspired perhaps by the OpenZika platform, which WHO helped create to encourage researchers to share their scientific knowledge about the Zika virus in real time in 2016). A concrete yet different example that stimulates and advances the work to suppress the COVID-19 pandemic is the COVID-19 Vaccines Global Access—or COVAX—a global initiative aimed at equitable access to COVID-19 vaccines led why the WHO, together with the Global Alliance for Vaccines and Immunization, the Coalition for Epidemic Preparedness Innovators, and other global health actors.

Now take function (*i*) to promote the improvement of nutrition, housing, sanitation, recreation, economic or working conditions, and other aspects of environmental hygiene. Function (i), as mentioned above, is the source of what today WHO calls the SDH. For the sake of simplicity, I will elect one single aspect of function (i), namely to promote the improvement of recreation. There is little doubt that recreation is a human good, necessary for our health and well-being. Few people would disagree with the necessity of leisure for attaining good health and human flourishing. However, when the WHO lists together function (g), which would justify, for example, the crucial access to medical knowledge on COVID-19 vaccines, and function (i) based on the human need of recreation, WHO creates the impression that both functions are *prima facie* and *ceteris paribus* on a moral par for the purpose of their priority setting. But they are not equal priorities for the WHO in a context of scarce resources and limited capacities—which is WHO’s reality. Because they are not equal priorities for the WHO, they should not receive the same amount of funding or the same level of institutional support, coming from the WHO.

To list functions (g) and (i) together, with no clear distinction regarding how WHO should set priorities when capacity to discharge both functions is insufficient, inevitably creates practical difficulties and confusions that could be avoided by conceptual clarifications. Access to medical knowledge on COVID-19 vaccines [function (g)] pertains to the realm of basic health needs that qualify as PHEIC. Recreation [function (i)] belongs to the category of nonbasic health needs/SDH. These distinctions are relevant and should be made explicit, if confusion is to be avoided. Again, this is because the different rights and duties that bear on these two different aspects of global health justice, namely the basic and nonbasic needs of health, are not so straightforwardly discerned in practice—particularly within a very pressing context of global scarcity, coupled with WHO’s overcommitments and overextensions, where WHO is pulled in different directions. And all of these make the distinction between priority and nonpriority a difficult and confusing enterprise for the WHO to make on the spot, during a global crisis (WHO, 2013a: 16; WHO, 2013b: para 50).

One could rightly object here and say that recreation is vital, including in an exceptional context of emergency like the COVID-19 pandemic. The objector here could bring me the example of healthcare professionals, caring for COVID-19 patients: they need adequate leisure to recharge their energies and be able to continue to care for their patients. Then, the objector could also rightly remind me that recreation has perhaps become even more relevant during the COVID-19 lockdowns, because of the pandemic’s high impact on mental and physical health. And I would agree with my objector here: (a) it is irrefutable that healthcare professionals have a right to rest and also a duty to care for themselves adequately; and (b) it is also unquestionable that recreation is indispensable for an adequate level of mental and physical health. However, these do not entail that, in an exceptional context of emergency like the COVID-19 pandemic, the WHO should be as committed to recreation as it is in a context of normalcy, where scarcity and limitations are not as pressing. In fact, it is hard to imagine how recreation would not be somehow de-prioritized by the WHO (at least in the short-term) during a pandemic. This is not to say that individuals, local communities, governments should not try to use their creativity and foster forms of recreations during times of crisis (after all, recreation is necessary to build resilience and hope in times of difficulties). But this is just to say that WHO, pressed by time and resource constraints, may need to put considerations regarding recreation (together with other nonbasic health needs/SDH and basic needs that do not qualify as PHEIC) aside in order to be able to discern clearly how to set priorities and ration scarce healthcare resources in responding to the pandemic. Again, this does not mean that recreation (or any other nonbasic health needs/SDH and non-PHEIC basic health needs) is not important. Quite the contrary: recreation is crucial for physical and mental health. But my point is that the WHO’s mission to promote recreation should be delegated to national governments and local communities, instead of being imposed from the top-down (i.e., from the WHO to national governments and local communities, who are better placed to design safe COVID-19 policies on recreational activities, attuned to their local costumes).

Recreation and other nonbasic health needs/SDH will, more directly or more indirectly, influence people’s health. Although these nonbasic health needs/SDH are all relevant (some way or another) to individual and population health, they cannot be all reduced to health, because they often need to be addressed in particular ways. To put this another way, the right to health is not the only human right (Tasioulas, 2020). Sure, human rights are all interdependent and indivisible, meaning that they cannot be enjoyed fully without another. However, human rights cannot be all reduced to an all-encompassing reading of the right to health (Tasioulas, 2020). Recreation is a distinct right, which should be the priority of local public policies, local governments, and local communities, rather than the priority of the WHO. Locals should be primarily in charge of recreation (and other nonbasic health needs/SDH), unless the local government and local communities have requested WHO’s (and other countries and other global health stakeholders’) assistance in respecting, protecting, and fulfilling the right to recreation. Again, I am not suggesting here that recreation is unconnected to health. Recreation bears on health. Good health depends on recreation. But health and recreation are irreducible human goods: both are necessary for the good life of an individual and the common good of all. And a human good cannot be reduced as mere means to another human good (Finnis, 1983). To reduce recreation to health and to conflate these two human goods is a mistake: each ground different rights, and each of these rights will ground different duties for their full realization.

To set aside recreation in order to free WHO to focus the use of its scarce resources on tackling core global emergency questions pertaining a pandemic is, in other words, to morally distinguish different aspects of the human right to health (i.e., PHEIC basic health needs, non-PHEIC basic health needs, and nonbasic health needs/SDH). This exercise of moral reasoning is, obviously, not always straightforward. The clear separation between basic and nonbasic health needs/SDH (de Campos, 2016), and between basic health needs that qualify as global health emergencies and those basic health needs that are not PHEIC (de Campos, 2020), is not always evident, requiring some moral contemplation that allows for careful analysis. But thorough moral reasoning requires time. So the moment when an outbreak emerges and difficult ethical decisions need to be made quickly and in coordination with other countries to contain the spread of infectious diseases is not the best time for engaging with nuanced moral reasoning of this sort. The most appropriate time for moral contemplation of this kind, leading to clear moral distinctions that provide good guidance and reasons for action, is *before* another outbreak occurs and not *during* a global crisis. The WHO reform, which will likely take place in the aftermath of the COVID-19 pandemic, offers a good opportunity to review the WHO constitution, in general, and WHO’s overambitious mandate reflecting WHO’s inflated definition of health as well-being, in particular. In this review process, WHO would do well in identifying the core global emergency questions which the organization should prioritize for now, delegating most of the non-PHEIC basic health needs and the nonbasic health needs/SDH to other global health stakeholders, better suited to realize them.

My objector here would want to remind me and insist that the well-being conception of the right to health and WHO's broad mandate are not necessarily inimical to priority setting: the well-being conception of the right to health and WHO's broad mandate can, my opponent would argue, appreciate for example that the pursuit of recreation and the pursuit of a vaccine for COVID-19 are, naturally, priorities of different status. Although recreation and a COVID-19 vaccine should not receive the same amount of funding or institutional support, there are—my opponent would maintain—good reasons to have a broad range of foci under the WHO remit. For example, a broad mandate with several functions, would allow sharing of expertise between areas, and my overly literal interpretation of a list of priorities would impede such organizational sharing.

True, sharing and coordination are one of WHO’s core purposes: as the coordinating authority for global health, WHO is indeed expected to share communication in a coordinated manner within the WHO itself and with other global stakeholders. But my point is exactly that proper sharing and coordination have not happened. WHO has not been successful in performing its core purposes. And one of the reasons why WHO has not adequately shared communication in a coordinated manner is moral conflation and the overcommitments that ensue.^11^

The moral conflations embedded in the WHO constitution are not merely a theoretical abstract predicament to entertain philosophical minds. These moral conflations have serious practical consequences. The lack of a more nuanced understanding of WHO’s mandate and a more sophisticated conception of the human right to health—nuanced and sophisticated enough to capture the moral distinction between basic and nonbasic health needs/SDH (de Campos, 2016), and between global health emergencies and basic health needs that arise outside of PHEIC (de Campos, 2020)—inevitably leads to moral confusions.

Moral confusion is the source of practical indecision, then leading to either inaction or imprudent action. And these have been precisely the upshots of WHO’s moral confusion: inaction and imprudent action, especially in the context of PHEICs, such as the Ebola and COVID-19 outbreaks, when WHO’s delays and unwise choices have been heavily criticized. In trying to do everything that its mandate allows it to do, WHO has been unable to prioritize what needs to be done first. Morally confused, WHO has lost its bearings, either in its inaction or in its imprudent actions. In its own words, WHO has “spread too thinly” (WHO, 2013a). How to reform the WHO, though? I here suggest a dose of humility to counteract overambition.

## The Humble Approach to WHO Reform

The expansive approach toz the WHO reform conflates good leadership in global health governance with the ambition to attain for all peoples the (impossible) standard of complete health (i.e., “state of complete physical, mental, and social well-being and not merely the absence of disease or infirmity”^12^), as defined in the WHO constitution and subsequent UN documents establishing the human right to health. This study has discussed the underlying moral problems of the expansive approach as well as its practical implications. In this section the study puts forth its central argument that to be a better leader, WHO should be less ambitious, do much less, and have a much narrower mandate. The humble approach to the WHO reform not only makes sense in terms of (i) political feasibility, but also in terms of (ii) ethical reasons. First, it is politically unfeasible to expect a dramatic expansion of WHO’s budget, as it would be in fact required if WHO were to fulfill all or most of the twenty-two functions listed in WHO’s constitution. Second, it is ethically unreasonable to keep WHO responsible for performing those functions that would be better performed by other stakeholders, especially governments and local actors, which are more cognizant of the particularities of their epidemiological conditions, institutional culture, and social traditions.^13^ Given the political unfeasibility and the ethical unreasonableness of the expansive approach to the WHO reform, a better approach would be to do less and delegate more of WHO’s functions to other global health stakeholders (especially governments and local actors). In this way, the functions that WHO itself performs would be restricted to those that can be best performed by WHO itself, rather than other global health stakeholders (Clift, 2014: 12).

What are the roles that the WHO is uniquely placed to execute? As discussed in the previous section, WHO’s mandate and functions are very broad. WHO itself has acknowledged that it “lacks a clear grasp of its comparative advantage, including at country level, at times taking on what others might do better” (WHO, 2013a). Global health experts have tried to identify WHO’s core functions amidst those 22 listed in article 2 of the WHO constitution. Some experts have reduced the lengthy list to nine core functions, although they could not all agree on the nine items (Clift, 2014: 9). Others have defined the four essential functions of the global health system, and one would expect that these four items would be more agreeable or less contentious (Frenk and Moon, 2013: 940). Yet others have managed to further simplify the list and more accurately identify the two core functions of the WHO: (i) its technical role of knowledge generation and dissemination, in which WHO has the responsibility to gather and share epidemic intelligence; and (ii) normative role of knowledge translation, in which the WHO has the responsibility to translate scientific evidence into advice and recommendations on how to control a PHEIC (Lee and Walt, 1992; Lee and Pang, 2014: 120).^14^

In the post COVID-19 scenario, it is expected that there will be little disagreement on the need for better coordinated communication of available knowledge to prevent and respond to global health threats like pandemics. In this way, most people would agree that the functions of (i) knowledge generation and dissemination and (ii) knowledge translation are indeed WHO’s two core functions, given their indispensable role in controlling PHEICs. These two functions, therefore, establish the common ground on which most people can agree when it comes to WHO functions. However, in order to generate, disseminate, and translate knowledge on PHEIC and do it not only properly, but *well*, WHO would need to free itself of other nonbasic and nonemergency-related functions. This would enable WHO to focus the use of its scarce resources on ensuring that coordinated communications regarding PHEIC are truthful, evidence-based, consistent, and timely shared among all WHO member-states and other global health stakeholders.

There is, nevertheless, a complication with the humble approach to the WHO reform and the required delegation of WHO functions to other global health stakeholders. The complication is this: since WHO’s budget depends in large proportion on donations, and donors normally tend to tie their donation to the performance of specific functions, tasks, and programs (Lee and Walt, 1992; Lee and Pang, 2014: 120),^15^ the decision to delegate particular functions to other stakeholders may not necessarily result in additional resources that would then be free to be allocated for the retained functions (Clift, 2014: 11). This is indeed a difficulty. However, the fact that something is challenging does not entail that it is not the right thing to do, or that it should not be pursued. Truthful communication and further negotiations with donors could quite plausibly lead to a satisfactory outcome enabling the humble approach to WHO reform. This would be a credible scenario if donors were convinced of the benefits of the humble approach.

Donors would have to be persuaded of the advantages of drastically reducing WHO’s mandate and solely focusing on gathering scientific evidence on how to control PHEIC and communicating it in a truthful, timely, and coordinated manner. This humble mandate for the WHO proves beneficial and reasonable in at least two (interconnected) ways. First, by focusing exclusively on ensuring truthful, timely, and coordinated communications among stakeholders, WHO would be able to concentrate its finite capacities in performing well its key purpose as a coordinating body for global health threats like pandemics. The benefit here would be a much-needed enhanced effectiveness in global health coordination and communication in the context of PHEIC.

Second, in performing said coordinated communications well, WHO could potentially regain public trust as a legitimate coordinating authority for global health security matters.^16^ During the COVID-19 pandemic, concealment of information, misinformation, and incoherent communication have further eroded trust in WHO (Friedman, 2020).^17^ For example, inconsistent advice and changing messages about the personal use of masks without previous clarification on why the issue was divisive, gave the public the impression that decisions by the WHO and governments on this matter were unreliable and arbitrary (Yong, 2020). The changing nature of COVID-19 policies on masks was based on emerging scientific evidence of asymptomatic viral transmission, which recommended that everyone wear a face mask in public spaces. In other words, the shifting scientific evidence grounded the changing guidance in COVID-19 policies. The problem, however, was that this communication process between the scientific community and policymakers was not always accurately shared with (i.e., contextualized and explained to) all other stakeholders, including the public, and this led to the erosion of public trust in health authorities like the WHO (de Campos-Rudinsky and Undurraga, 2021; Veit *et al.*, 2021). A similar pattern of inconsistent recommendation and shifting messages was also observed in relation to travel bans: while not instructing countries against quarantines, WHO advised countries against travel bans pursuant to IHR.^18^ But governments subsequently established travel bans together with quarantines, disregarding the binding legal document (i.e., the IHR) to which they had agreed. Perhaps governments would have been more compliant with WHO’s directions on travel restrictions if WHO had done a better job not only in fully contextualizing why the issue was divisive, but also in explaining the evidence-based reasons why WHO advised against travel bans while not discouraging quarantine at the same time. WHO failed to accurately communicate why their advice on travel restrictions and quarantines were not necessarily contradictory, and how they are actually consistent within the IHR framework (Habibi *et al.*, 2020).

Accurate communication means communication that is truthful, evidence-based, consistent, and timely shared*.* This requires being transparent about what WHO, governments, and scientists know and do not yet know about the virus and the disease. When decision-makers are truthful about what they know and do not know, and are truthful about uncertainties, the public can better understand when shifting evidence leads to a new policy direction without forming the impression that those changes are whimsical or defective (Yong, 2020).^19^ However, this level of honesty about what one does not know necessitates humility in acknowledging one’s limitations. Although humility in accepting one’s vulnerabilities in this way is uncomfortable, it is highly effective in building trustworthiness. This is precisely the second benefit of the humble approach: it has the potential to redeem the public trust in WHO’s words and deeds.^20^

## Conclusions

This study argued against the expansive approach to the WHO reform, according to which to be a better global health leader, WHO should do more, be given more power and financial resources, have more operational capacities, and have more teeth by introducing more coercive monitoring and compliance mechanisms to its IHR (Gostin, 2014, 2020; Gostin and Friedman, 2014; Gostin *et al.*, 2015; Kickbusch and Reddy, 2015; Mackey, 2016; Negri, 2018; Burci, 2020; Gostin and Wetter, 2020). The main flaw of the expansive approach is to conflate good leadership in global health governance with the ambition to attain for all peoples the (impossible) standard of complete health (i.e., “state of complete physical, mental, and social well-being and not merely the absence of disease or infirmity”^21^), as defined in the WHO constitution and UN documents establishing the human right to health, which is neither politically feasible nor morally justifiable.

As an alternative, the study put forth the humble approach to the WHO reform, and argued that to be a better leader, WHO should do much less and have a much narrower mandate. More specifically, WHO should focus exclusively on coordination efforts, by ensuring truthful, evidence-based, consistent, and timely shared communications regarding PHEIC among WHO member-states and other global health stakeholders, if the organization desires to be a real global health leader whose authority the international community respects and whose guidance people trust.

## Footnotes

1 For a historical analysis of the perennial nature of WHO reform debates since the 1990s, see (Lee and Pang 2014).

2 WHO, International Health Regulations (IHR), 2005, WHA 58.3, 2nd edn. Geneva: World Health Organization

3 WHO Constitution, 1948, Preamble

4 WHO Constitution, 1948, Preamble. See also: UN International Covenant on Economic, Social and Cultural Rights (ICESCR), 1966, art 12; and UN Committee on Economic, Social and Cultural Rights (CESCR) *General comment No. 14: The Right to the Highest Attainable Standard of Health (Art 12 of the International Covenant on Economic, Social and Cultural Rights)*, 2000, E/C.12/2000/4, 12 August.

5 See: http://www.who.int/social_determinants/en/.

6 On justice-related questions on social determinants of health, including the relationship among income, inequality, and heath, see the 2009 special symposium edition on “Justice and the Social Determinants of Health” of this journal, in particular: (Venkatapuram, 2009; Verweij, 2009; Wilson, 2009; Wolff, 2009). On the legal aspects of social determinants of health, see: (Coggon 2020)

7 WHO Constitution, 1948, Preamble

8 WHO Constitution, 1948, Art 1.

9 WHO Constitution, 1948, Preamble. See also: UN International Covenant on Economic, Social and Cultural Rights (ICESCR), 1966, art 12; and UN Committee on Economic, Social and Cultural Rights (CESCR) *General comment No. 14: The Right to the Highest Attainable Standard of Health (Art 12 of the International Covenant on Economic, Social and Cultural Rights)*, 2000, E/C.12/2000/4, 12 August.

10 For a detailed discussion of the problem of moral conflation in the well-being conception of health, see: de Campos, Cambridge University Press, 2017, ch 1, p.20.

11 Coordination and communication are not synonymous. Coordination is WHO’s core purpose and communication is a condition for coordination to ensue. But if truthful and timely communications are necessary conditions for good coordination, then by focusing on ensuring truthful and timely communications among stakeholders, WHO would be focusing on performing its core purpose (i.e., coordination) well.

12 WHO Constitution, 1948, Preamble. See also: UN International Covenant on Economic, Social and Cultural Rights (ICESCR), 1966, art 12; and UN Committee on Economic, Social and Cultural Rights (CESCR) *General comment No. 14: The Right to the Highest Attainable Standard of Health (Art 12 of the International Covenant on Economic, Social and Cultural Rights)*, 2000, E/C.12/2000/4, 12 August.

13 This would be contrary to the structural principle of subsidiarity. The principle of subsidiarity has proved to be an effective governance tool over centuries. It basically establishes that where families, neighborhoods, and local communities, and local governments can effectively address their own problems, they should do so; and only where they cannot, then other countries and other higher-level structures of power and authority should intervene and provide aid (i.e., *subsidium*). The term “sub-sid-iary”—which literally means to “seat” (“sid”) an activity down (“sub”) as close to the problem as possible—recognizes the value of first trying to solve social problems locally and moving up to higher levels of governance only as necessary. See: de Campos, 2017, chapter 3.

14 More specifically, these two functions would include, for example, collecting and disseminating international health data, agreeing nomenclature, producing guidelines and protocols, promoting research, hosting technical meetings of experts.

15 Examples of donor-friendly activities whose impact is more visible, include: providing emergency relief, distributing drugs,, immunizing children, and fighting outbreaks on the ground. These are more impactful because they tend to more easily to generate public approval. (Lee, Pang 2014, at 120)

16 On the relationship between truthful communications and trust (or lack thereof) in the WHO, see: Prah Ruger 2020. For a philosophical account on the relationship between truthfulness and trust, see: O’Neill, 2002. For O’Neill, to deserve trust, healthcare institutions and practitioners must fulfil the requirements of trustworthiness, which include honesty, particularly in their communications. On the relationship between truthfulness and trust in public health institutions, such as the WHO, in the particular context of the COVID-19 pandemic, see: Veit, Brown, Earp, 2020; van der Bles, van der Linden, Freeman, Spiegelhalter, 2020.

17 Recent events during the COVID-19 pandemic have led some to believe that there is a widespread lack of trust not only in international cooperation and multilateralism in general, but in the WHO in particular (Taylor; Habibi. 2020; Gvosdev 2020). The erosion of public trust in the WHO is an ongoing problem. (Jha 2017; Bain, Ebuenyi 2017). More recently, the lack of trust in the WHO was manifested itself more concretely, for example, in the US withdraw from the WHO under the Trump administration, Brazil’s withdraw from the organization under Bolsonaro’s administration, as well as in several other countries’ complete disregard of WHO’s recommendations for the COVID-19 pandemic suppression (including WHO’s legal recommendations and obligations contained in International Health Regulations, the multilateral instrument that governs how 196 states and WHO should collectively contain the global spread of diseases). (Gilsinan, 2020; Habibi *et al.*, 2020; Victor and Hauser 2020).

18 Article 43 of the IHR restricts the measures that WHO member-states can implement when addressing public health threats when those measures are not supported by science, commensurate with the risks involved, or anchored in human rights. (WHO. 2005. International Health Regulations, WHA 58.3, 2nd edn. Geneva: World Health Organization, Art 43). The purpose of article 43 is to prevent member-states from taking unnecessary measures that may harm people or that dis-incentivize other countries from reporting new public health threats to international public health authorities. (Habibi *et al*., 2020)

19 I have used the word “truthful” throughout this paper to mean honest, open, candid, transparent communications, especially about one’s limitations (e.g., institutional limitations, capacity limitations, knowledge limitations). My use of the word ‘truthful’ should not imply, however, that the WHO (or other public health authorities) certainly communicated lies in the past, casting aspersions on the legitimacy of the WHO as an agency.

20 Flawed communication *alone* may not justify a complete institutional reform of the WHO. This would be stretching the argument presented in this paper. Nevertheless, lack of truthful, timely, and coordinated communications combined with the erosion of public trust in the organization are strong indications that *some* sort of institutional reform is necessary and would be welcomed. All that the paper is arguing is that, if WHO would commit to do less, openly sharing what the organization can and cannot do (including what the organization has done well since 1946), the organization could then also address the problem of erosion of public trust. This is the sort of reform (which I have called the humble approach) that this paper has argued for and the potential benefits that it could bring.

21 WHO Constitution, 1948, Preamble. See also: UN International Covenant on Economic, Social and Cultural Rights (ICESCR), 1966, art 12; and UN Committee on Economic, Social and Cultural Rights (CESCR) *General comment No. 14: The Right to the Highest Attainable Standard of Health (Art 12 of the International Covenant on Economic, Social and Cultural Rights)*, 2000, E/C.12/2000/4, 12 August
